# Tepotinib in patients with NSCLC harbouring *MET* exon 14 skipping: Japanese subset analysis from the Phase II VISION study

**DOI:** 10.1093/jjco/hyab072

**Published:** 2021-05-25

**Authors:** Hiroshi Sakai, Masahiro Morise, Terufumi Kato, Shingo Matsumoto, Tomohiro Sakamoto, Toru Kumagai, Takaaki Tokito, Shinji Atagi, Toshiyuki Kozuki, Hiroshi Tanaka, Kenichi Chikamori, Naofumi Shinagawa, Hiroaki Takeoka, Rolf Bruns, Josef Straub, Karl Maria Schumacher, Paul K Paik

**Affiliations:** 1 Department of Thoracic Oncology, Saitama Cancer Center, Ina, Japan; 2 Department of Respiratory Medicine, Nagoya University Hospital, Nagoya, Japan; 3 Department of Respiratory Medicine, Kanagawa Cancer Center, Yokohama, Japan; 4 Department of Thoracic Oncology, National Cancer Center Hospital East, Kashiwa, Japan; 5 Department of Respiratory Medicine, Tottori University Hospital, Yonago, Japan; 6 Department of Thoracic Oncology, Osaka International Cancer Institute, Osaka, Japan; 7 Department of Lung Cancer Center, Kurume University Hospital, Kurume, Japan; 8 Department of Thoracic Oncology, NHO Kinki-Chuo Chest Medical Center, Sakai, Japan; 9 Department of Respiratory Medicine, NHO Shikoku Cancer Center, Matsuyama, Japan; 10 Department of Internal Medicine, Niigata Cancer Center Hospital, Niigata, Japan; 11 Department of Oncology, NHO Yamaguchi—Ube Medical Center, Ube, Japan; 12 Department of Respiratory Medicine, Faculty of Medicine and Graduate School of Medicine, Hokkaido University, Sapporo, Japan; 13 Department of Respiratory Medicine, NHO Kyushu Medical Center, Fukuoka, Japan; 14 Department of Biostatistics, Merck KGaA, Darmstadt, Germany; 15 Translational Medicine, Department of Clinical Biomarkers and Companion Diagnostics, Merck KGaA, Darmstadt, Germany; 16 Global Clinical Development, Merck KGaA, Darmstadt, Germany; 17 Department of Medicine, Memorial Sloan Kettering Cancer Center, New York, NY, USA; 18 Department of Medicine, Weill Cornell Medical College, New York, NY, USA

**Keywords:** tepotinib, protein kinase inhibitors, proto-oncogene proteins c-Met, liquid biopsy, carcinoma, non-small-cell lung

## Abstract

**Background:**

*MET* exon 14 skipping is an oncogenic driver occurring in 3–4% of non-small cell lung cancer (NSCLC). The MET inhibitor tepotinib has demonstrated clinical efficacy in patients with *MET* exon 14 skipping NSCLC. Here, we present data from Japanese patients in the Phase II VISION study, evaluating the efficacy and safety of tepotinib.

**Methods:**

In the open-label, single-arm, Phase II VISION study, patients with advanced/metastatic NSCLC with *MET* exon 14 skipping received oral tepotinib 500 mg once daily. The primary endpoint was objective response by independent review. Subgroup analyses of Japanese patients were preplanned.

**Results:**

As of 1 January 2020, 19 Japanese patients received tepotinib and were evaluated for safety, 15 of whom had ≥9 months’ follow-up and were also analysed for efficacy. By independent review, objective response rate (ORR) was 60.0% (95% confidence interval [CI]: 32.3, 83.7), median duration of response was not reached (95% CI: 6.9, not estimable [ne]), and progression-free survival was 11.0 months (95% CI: 1.4, ne). ORR in patients with *MET* exon 14 skipping identified by liquid biopsy (*n* = 8) was 87.5% (95% CI: 47.3, 99.7), and by tissue biopsy (*n* = 12) was 50.0% (95% CI: 21.1, 78.9). Patients’ quality of life was maintained with tepotinib treatment. Among patients evaluated for safety, the most common treatment-related adverse events (any grade) were blood creatinine increase and peripheral oedema (12 and nine patients, respectively).

**Conclusions:**

Tepotinib demonstrated robust and durable clinical efficacy in Japanese patients with advanced NSCLC harbouring *MET* exon 14 skipping, identified by either liquid or tissue biopsy. The main adverse events, blood creatinine increase and peripheral oedema, were manageable.

## Teaser

In VISION, tepotinib demonstrated robust efficacy in Japanese patients with *MET* exon 14 skipping NSCLC. The safety profile was tolerable, with mostly mild–moderate adverse events and few treatment discontinuations.

## Introduction

Small-molecule tyrosine kinase inhibitors targeting oncogenic driver mutations (such as *EGFR*, *ALK*, *ROS1* and *BRAF* (1)) have fundamentally changed the treatment paradigm for non-small cell lung cancer (NSCLC). As a result, the Japanese Lung Cancer Society recommends molecular testing to identify biomarkers such as driver oncogenes, as well as the use of kinase inhibitors as first-line treatment in patients with NSCLC harbouring oncogenic driver mutations (2).

Alterations in the *MET* gene have recently been identified as oncogenic driver mutations in NSCLC (3), and correlate with poor patient survival relative to patients with NSCLC expressing wild-type *MET* (4)*. MET* exon 14 skipping is an alteration in the *MET* gene that can lead to MET dysregulation, which occurs in 1–4% of East Asian patients with NSCLC (5–7) and 3–4% of Western patients (8–10). This alteration can be detected by analysing circulating tumour DNA (ctDNA) in plasma obtained from liquid biopsy samples, or through analysing RNA/DNA from tissue biopsy samples. Compared to patient populations defined by other actionable alterations, patients with *MET* exon 14 skipping are typically older, more evenly distributed by sex, and have a greater proportion of current/former smokers (10,11)*.*

Tepotinib is a once daily, orally available, highly selective and potent MET tyrosine kinase inhibitor (12,13) that was approved in March 2020 by the Japanese Ministry of Health, Labour and Welfare for the line-agnostic treatment of patients with unresectable advanced or recurrent NSCLC with *MET* exon 14 skipping. Tepotinib was approved in this setting based on results from the Phase II VISION study (14). VISION (NCT02864992) is a global, single-arm study of tepotinib in patients with advanced NSCLC harbouring *MET* exon 14 skipping (Cohorts A and C). Cohort A has completed enrollment and reported an objective response rate (ORR; primary endpoint) across treatment lines of 46.5% by independent review, and 55.6% by investigator assessment; confirmatory analysis will be conducted in Cohort C (15). The ORR for tepotinib was consistent between patients with *MET* exon 14 skipping identified by either liquid or tissue biopsy (48.5% vs 50.0%); the median duration of response (DOR) was 11.1 months, and the onset of response mostly occurred within 6 weeks of tepotinib treatment initiation. Tepotinib is well tolerated in NSCLC patients, with most treatment-related adverse events (AEs) being of Grade 1 or 2, and few leading to treatment discontinuations (15).

Here, we report efficacy and safety data for Japanese patients with advanced NSCLC harbouring *MET* exon 14 skipping who were enrolled in the Phase II VISION study.

## Methods

### Study design and objectives

The ongoing VISION study (NCT02864992) is a Phase II, single-arm, open-label, multicentre trial conducted in >130 sites across 11 countries, including 13 sites in Japan. The study aims to assess the antitumour activity and tolerability of 500 mg tepotinib given orally immediately after a meal, once daily until disease progression, consent withdrawal or AEs leading to discontinuation in patients with advanced NSCLC harbouring *MET* exon 14 skipping.

The study was conducted in accordance with the Declaration of Helsinki, International Conference on Harmonisation Good Clinical Practice, local laws, and applicable regulatory requirements. The study was funded by the sponsor, who was responsible for the collection and analysis of the data, and had a role in data interpretation. All patients provided written informed consent for participation and the study was approved by the institutional review board or independent ethics committee of each centre.

### Patients

Eligible Japanese patients were aged 20 years or older with histologically or cytologically confirmed advanced (locally advanced or metastatic) NSCLC, measurable disease per Response Evaluation Criteria in Solid Tumors (RECIST) v1.1, and Eastern Cooperative Oncology Group performance status 0 or 1. Patients could enrol in the study based on results from either liquid or tissue biopsy, reflecting how testing may occur in clinical practice and enabling a larger patient population to benefit.

*MET* exon 14 skipping was detected centrally by next-generation sequencing panels Guardant360® (73-gene) and Oncomine™ Focus Assay (52-gene, Thermo Fisher Scientific, Waltham, MA, USA), which analysed ctDNA and tumour tissue RNA, respectively. ctDNA analysis detected alterations occurring in the splice acceptor or donor regions of *MET* exon 14, known to lead to skipping. RNA analysis involved a functional assay to directly demonstrate *MET* exon 14 skipping occurring at the level of gene expression.

Patients could also be enrolled on the basis of tissue biopsy results (using real-time polymerase chain reaction methodology described by Sunami et al.) at LC-SCRUM, a nationwide cancer genomic screening project for the application of personalised medicine to advanced NSCLC (16,17). Central confirmation of *MET* exon 14 skipping that was identified by LC-SCRUM was not required before patient enrolment.

Patients could have had no more than two lines of prior treatment for advanced/metastatic disease (prior MET inhibitors were not permitted; immunotherapy was allowed), and patients with tumours harbouring activating *EGFR* mutations or *ALK* rearrangements were excluded.

### Study endpoints and assessments

The primary endpoint was objective response (defined as confirmed complete or partial response) determined according to RECIST v1.1, based on independent review. Confirmation was obtained by a tumour assessment at least 4 weeks (28 days) after the initial tumour assessment indicating complete or partial response. Secondary endpoints included investigator-assessed objective response, DOR, progression-free survival (PFS), overall survival (OS), patient-reported outcomes, and safety.

Efficacy assessments and patient-reported outcome evaluations were conducted every 6 weeks within the first 9 months of treatment (and every 12 weeks thereafter). Tumours were assessed by computed tomography (CT) or magnetic resonance imaging of the chest, abdomen and pelvis. Additional anatomic areas were investigated based on individual signs or symptoms. Patients’ quality of life was assessed by using the European Organisation for Research and Treatment of Cancer Quality of Life Lung Cancer-13 questionnaire (EORTC QLQ-LC13) (18), EORTC QLQ Core 30 (EORTC QLQ-C30) (19), and the EuroQol Five Dimension Five Level Scale (20). Safety was evaluated using clinical laboratory tests and physical examination. AEs were graded according to the National Cancer Institute Common Terminology Criteria for Adverse Events v4.03.

Blood samples for exploratory biomarker ctDNA analyses were obtained at baseline, at Weeks 6, 12, and at the end of treatment, and were tested with the Guardant360® next-generation sequencing panel. A molecular ctDNA response to tepotinib was defined as either complete (100% depletion of *MET* exon 14 alterations in ctDNA indicating no detection of the *MET* exon 14 variant) or deep (>75% but <100% depletion) (21).

### Statistical analysis

No formal statistical comparisons were conducted; data were analysed in a descriptive manner. DOR, PFS and OS were analysed using Kaplan–Meier methods.

The safety population comprised all patients enrolled in the study who received ≥1 dose of tepotinib. Patients who had at least 9 months’ follow-up were analysed for efficacy. Japanese patients (enrolled in Japan) were analysed as a predefined subset.

## Results

### Patients

As of 1 January 2020, 6708 patients had been pre-screened for eligibility into the VISION study and 169 patients screened for inclusion into Cohort A. One hundred and fifty-two patients received at least one dose of tepotinib, including 19 patients enrolled in Japan; these patients were assessed for safety. Fifteen Japanese patients had ≥9 months’ follow-up at the time of analysis and were assessed for efficacy.

The median age of Japanese patients evaluated for efficacy (*n* = 15) was 69.4 years (range 64–82), patients were mostly male (60.0%), and adenocarcinoma was the main histologic subtype (93.3%) (full baseline characteristics are shown in Table 1). Baseline characteristics were consistent between patients enrolled by liquid or tissue biopsy. Eight patients had received prior cancer treatment (including immunotherapy in four patients [nivolumab, *n* = 1; pembrolizumab, *n* = 3]). The best overall response to last anticancer therapy in previously treated patients was one complete response and one partial response, with the median DOR being 5.5 months (range 3.0–8.0). Median PFS with last anticancer therapy was 4.5 months (range 3.0–12.0). The median duration of tepotinib treatment was 10.4 months (range 0.3–24.7), and as of 30 November 2020, four Japanese patients were still receiving treatment with tepotinib.

**Table 1:** Baseline characteristics

	Japanese patients^c^ *n* = 15
Age, median, years (range)	69.4 (64–82)
Aged <75 years, *n* (%)	11 (73.3)
Male, *n* (%)	9 (60.0)
Smoking history^a^, *n* (%)	
Ever used	8 (53.3)
Never used	6 (40.0)
ECOG performance status score, *n* (%)	
0	6 (40.0)
1	9 (60.0)
Histological subtype, *n* (%)	
Adenocarcinoma	14 (93.3)
NSCLC not otherwise specified	1 (6.7)
Number of lines of prior therapy, *n* (%)	
0	7 (46.7)
1	3 (20.0)
2+	5 (33.3)
Identification of *MET* exon 14 skipping, *n* (%)	
Liquid biopsy	8 (53.3)
Tissue biopsy^b^	12 (80.0)

^a^Smoking history data were missing for one patient.

^b^Eight patients with *MET* exon 14 skipping identified by tissue biopsy were enrolled via LC-SCRUM; all patients enrolled via LC-SCRUM had retrospective confirmation of *MET* exon 14 skipping using the ArcherMET CDx assay.

^c^Eight patients enrolled by liquid biopsy testing had a median age of 68.9 (range: 66–82), four (50.0%) were male, five (62.5%) had a history of smoking, four (50.0%) had an ECOG performance score of 1, and four (50.0%) were treatment-naïve.

Twelve patients enrolled by tissue biopsy had a median age of 69.6 (range: 64–81), eight (66.7%) were male, six (50.0%) had a history of smoking, seven (58.3%) had an ECOG performance score of 1, and five (41.7%) were treatment-naïve.

ECOG, Eastern Cooperative Oncology Group; NSCLC, non-small cell lung cancer.

### Efficacy

The ORR for Japanese patients with ≥9 months’ follow-up (*n* = 15) was 60.0% (95% confidence interval [CI]: 32.3, 83.7), as assessed by an independent review committee (IRC); nine patients had partial responses (Table 2). ORR in patients with *MET* exon 14 skipping identified by liquid biopsy (*n* = 8) was 87.5% (seven partial responses, 95% CI: 47.3, 99.7), and was 50.0% (six partial responses, 95% CI: 21.1, 78.9) by tissue biopsy (*n* = 12). ORR according to investigator assessment was 73.3% (95% CI: 44.9, 92.2) (10 partial responses and one complete response). Best overall response is shown in Table 2.

**Table 2:** Objective response rate, best overall response and median duration of response in efficacy-evaluable patients

	Japanese patients (*n* = 15)	
	IRC	Investigator
Objective response rate,	60.0	73.3
% (95% CI)	(32.3, 83.7)	(44.9, 92.2)
Best overall response, *n* (%)		
Complete response	0 (0.0)	1 (6.7)
Partial response	9 (60.0)	10 (66.7)
Stable disease	1 (6.7)	1 (6.7)
Progressive disease	4 (26.7)	2 (13.3)
Not evaluable	1 (6.7)	1 (6.7)
Median duration of response, months (95% CI)	ne (6.9, ne)	10.9 (3.2, ne)

CI, confidence interval; IRC, independent review committee; ne, not estimable.

**Figure 1.**
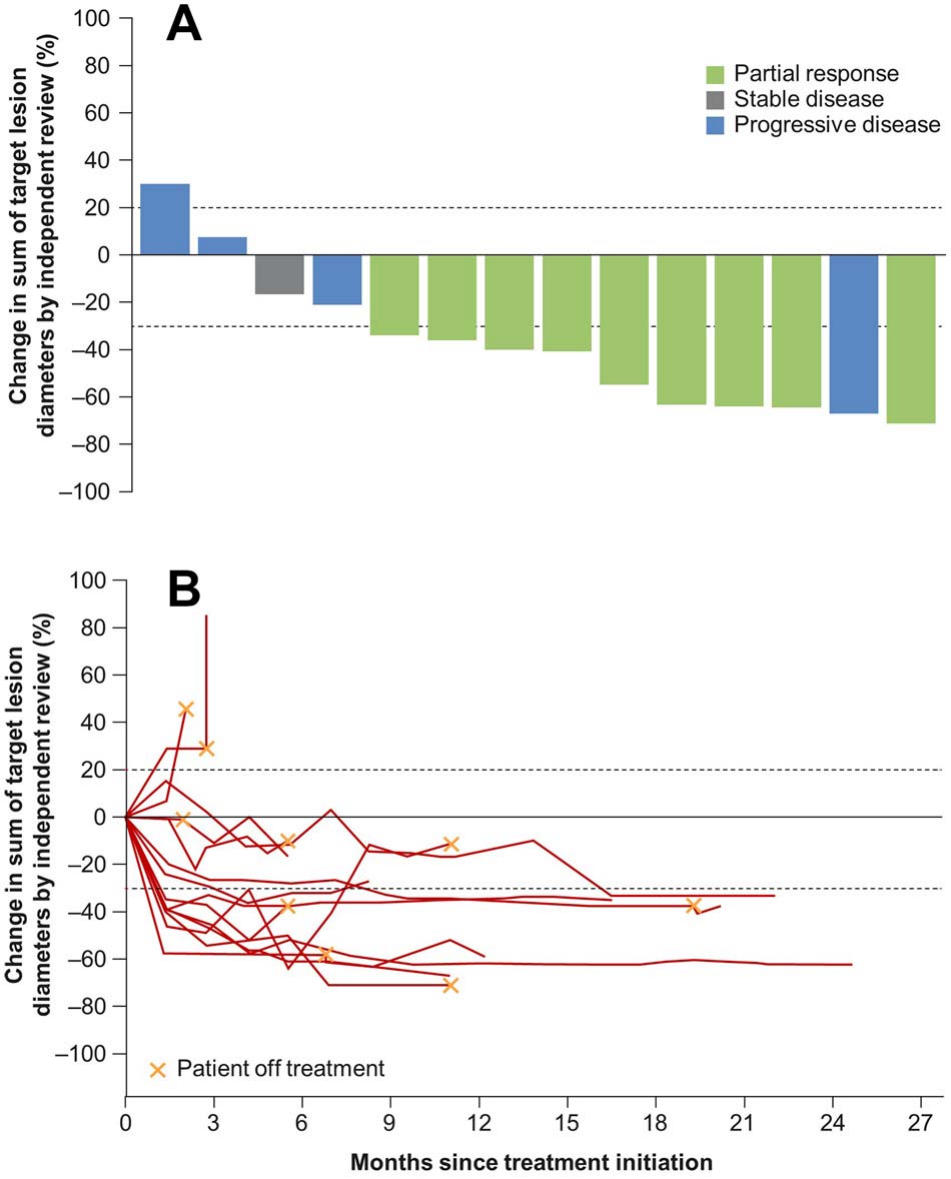
Antitumour activity in Japanese patients (*n* = 14). (A) Best percentage change in sum of longest diameters. (B) Change in sum of longest diameters over time. One patient died 10 days after treatment initiation due to pulmonary haemorrhage (not considered treatment-related) and, as such, change in tumour size is not available for this patient.

**Figure 2.**
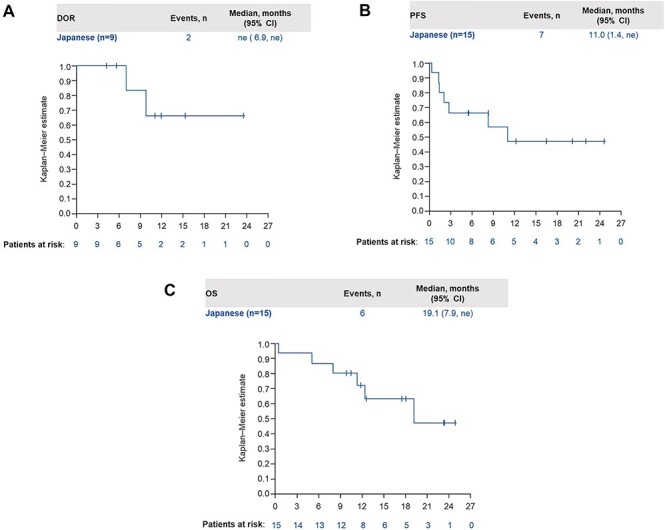
(A) DOR in Japanese patients by IRC. (B) PFS in Japanese patients by IRC. (C) OS in Japanese patients. CI, confidence interval; DOR, duration of response; IRC, independent review committee; ne, not estimable; OS, overall survival; PFS, progression-free survival.

Tumour shrinkage was observed in 12 patients (85.7%). Changes in tumour size for each patient with available data (*n* = 14) are shown in Fig. 1. The median DOR for patients with an objective response by IRC (*n* = 9) was not estimable (ne) (95% CI: 6.9, ne) and by investigator (*n* = 11) was 10.9 months (95% CI: 3.2, ne) (Fig. 2A). Median PFS by IRC was 11.0 months (95% CI: 1.4, ne), and by investigator assessment was 11.1 months (95% CI: 2.8, ne). Kaplan–Meier curves are shown in Fig. 2B. Median duration of follow-up for survival was 18.0 months (range 0.3–24.9) and median OS was 19.1 months (95% CI: 7.9, ne), although data are not yet mature (Fig. 2C). A case report of a Japanese patient whose tumour responded to tepotinib treatment with a long DOR is shown in Supplementary Fig. S1.

**Figure 3.**
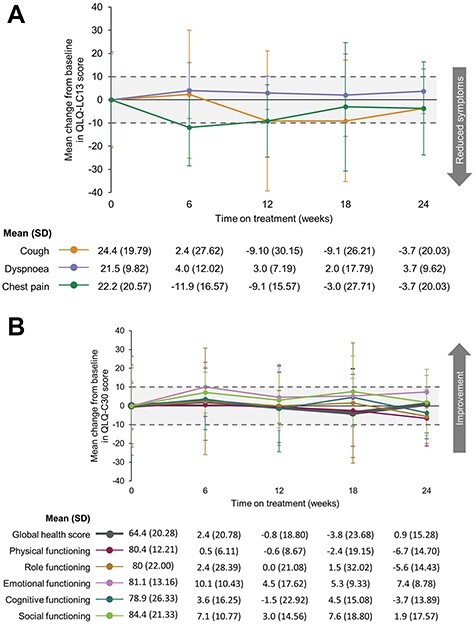
Quality of life on-treatment in Japanese patients: Mean change from baseline in patient-reported outcomes for (A) EORTC QLQ-LC13 symptom subscales and (B) EORTC QLQ-C30 global health score and subscales. An increase or decrease of >10 points was considered to be clinically meaningful (indicated with shaded area). All scores graded out of 100, with (A) lower = better and (B) higher = better. EORTC, European Organisation for Research and Treatment of Cancer; EORTC QLQ-LC13, EORTC Quality of Life Lung Cancer-13 questionnaire; EORTC QLQ-C30, EORTC QLQ Core 30; SD, standard deviation.

Among Japanese patients, completion rates for EORTC QLQ-C30 and EORTC QLQ-LC13 were 100% up to Week 24; nine patients remained on treatment at Week 24. Scores for global functioning showed stability in the patients’ reported quality of life over the 24-week analysis on the QLQ-C30 and QLQ-LC13 scales, with numerical improvements in cough and chest pain symptoms (Fig. 3).

Subsequent cancer therapy was received by seven Japanese patients (46.7%), most commonly atezolizumab, carboplatin, docetaxel and pemetrexed (either alone or in combination).

### Safety

Treatment-related AEs are summarized in Table 3. All Japanese patients who received tepotinib (*n* = 19) experienced an AE, and 18 patients a treatment-related AE. The most common treatment-related AEs were blood creatinine increase and peripheral oedema (12 and nine patients, respectively). Tepotinib treatment was interrupted in 13 Japanese patients (68.4%) due to treatment-related AEs, and dose reductions were reported in 10 patients (52.6%). Permanent discontinuation of treatment was reported in three patients (15.8%) due to interstitial lung disease (*n* = 1, Grade 2), diarrhoea and nausea (*n* = 1, Grade 2), and lung disorder (*n* = 1, Grade 1). Lung disorder was identified by bilateral ground glass patchy shadow in chest CT, was not associated with signs of infection, and resolved following treatment discontinuation without the need for steroids.

**Table 3:** Treatment-related AEs occurring in ≥15% of patients who received tepotinib

	Japanese patients (*n* = 19)	
Category, *n* (%)	All grades	Grade ≥ 3
Any treatment-related AE	18 (94.7)	9 (47.4)
Blood creatinine increased	12 (63.2)	0
Peripheral oedema	9 (47.4)	1 (5.3)
Diarrhoea	7 (36.8)	1 (5.3)
Amylase increased	5 (26.3)	1 (5.3)
Hypoalbuminemia	5 (26.3)	1 (5.3)
Lipase increased	4 (21.1)	1 (5.3)
Alanine aminotransferase increased	4 (21.1)	0
Aspartate aminotransferase increased	3 (15.8)	0
Dysgeusia	3 (15.8)	0
Nausea	3 (15.8)	0
Pruritus	3 (15.8)	0

AE, adverse event.

Peripheral oedema occurred in nine patients (47.4%) and Grade ≥ 3 oedema was observed in a low proportion of patients (*n* = 1, 5.3%). Gastrointestinal AEs, and changes in laboratory markers such as blood creatinine increase, hypoalbuminemia, and amylase and lipase increases were also common (Table 3).

Two cases of interstitial lung disease (Grades 1–2) that were considered by the investigator to be treatment-related were reported. One 76-year-old patient treated with tepotinib (first-line) for 207 days had Grade 2 interstitial lung disease after 21 days of treatment, which was managed with treatment interruptions and eventually led to treatment discontinuation. Another 67-year-old male patient treated with tepotinib (second-line) for 336 days had Grades 1 and 2 interstitial lung disease after 84 days of tepotinib treatment, which was managed with treatment interruptions and dose reductions. An independent review panel assessment found both patients had bilateral, moderate changes consistent with interstitial lung disease at baseline.

### Biomarker findings

All Japanese patients tested for *MET* exon 14 skipping by liquid biopsy had a corresponding on-treatment liquid biopsy sample and were evaluable for exploratory molecular response to tepotinib treatment (depletion of *MET* exon 14 skipping alterations detected in ctDNA).

**Figure 4.**
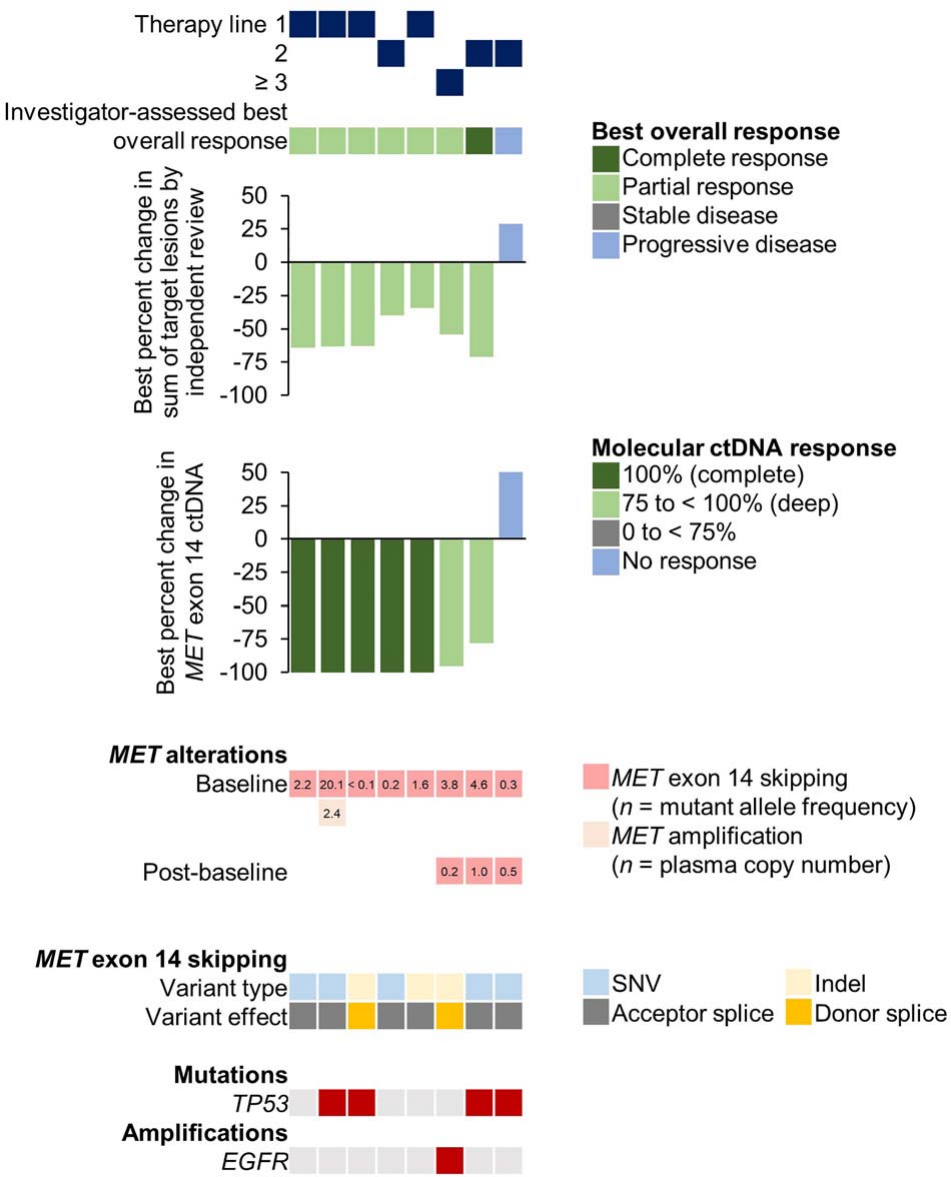
Best response to treatment and molecular response in Japanese patients with matched baseline and on-treatment liquid biopsy samples. ctDNA, circulating tumour DNA; Indel, insertion/deletion; SNV, single nucleotide variant.

A molecular response was observed in seven Japanese patients, with a corresponding radiographic response according to independent review also being observed in these patients (Fig. 4). One Japanese patient with progressive disease as best response did not demonstrate a molecular ctDNA response to tepotinib, but showed an increase from baseline in mutant allele frequency.

## Discussion

Tepotinib had durable clinical activity in Japanese patients with *MET* exon 14 skipping NSCLC, identified by liquid and/or tissue biopsy, with a response rate of 60.0% (*n* = 9/15) by IRC, a median DOR that was not reached, and a median PFS of 11.0 months. Median PFS was particularly encouraging given that the study population included patients in whom other courses of therapy had failed. Patient quality of life was also maintained during receipt of tepotinib. Molecular response to tepotinib corresponded with a favourable best overall response in patients with available data, indicating a potential role for liquid biopsy analysis in terms of translating molecular testing into clinical benefit for patients. Observed differences in response rate between patients enrolled by liquid or tissue biopsy should be interpreted with caution due to the small sample size of the Japanese subgroup. The response rate in Japanese patients with *MET* exon 14 skipping highlights the importance of molecular testing at initial diagnosis of advanced NSCLC, and is particularly relevant following the approval in Japan of a companion diagnostic using liquid or tissue biopsy samples for *MET* exon 14 skipping alterations.

Patients with *MET* exon 14 skipping NSCLC typically have a poor prognosis and low tumour response rates with non-targeted therapies. Two retrospective analyses of programmed cell death 1/programmed cell death ligand 1-directed immune checkpoint inhibitors in patients with *MET* exon 14 skipping NSCLC indicate poor response rates (ORR: 16–17%), although data specifically for Japanese patients were unavailable (22,23). In a retrospective study of East Asian patients with NSCLC, 18 patients with stage IV NSCLC harbouring *MET* exon 14 skipping who did not receive a targeted MET inhibitor achieved a median OS of 6.7 months (6).

The recent availability of targeted MET inhibitors has improved the outcomes for patients with NSCLC harbouring *MET* exon 14 skipping, although data in Japanese or Asian patients are relatively limited. The PROFILE 1001 study of the multi-kinase inhibitor crizotinib showed an ORR of 32% and a median PFS of 7.3 months in patients with *MET* exon 14 skipping NSCLC (24), and the co-MET study of crizotinib specifically in Japanese patients with NSCLC harbouring *MET* exon 14 skipping is ongoing (25). In a Chinese study of the selective MET inhibitor savolitinib in patients with *MET* exon 14 skipping NSCLC, ORR in the 61 patients who were evaluable for efficacy was 47.5% (26). Although in a different patient population, with *EGFR*-mutated NSCLC, preliminary data suggest savolitinib in combination with osimertinib, a third-generation EGFR tyrosine kinase inhibitor, is well tolerated in Japanese patients (27). In Japanese patients who received the selective MET inhibitor capmatinib for *MET* exon 14 skipping advanced NSCLC in the GEOMETRY mono-1 study, ORRs of 50.0% and 36.4% were observed for treatment-naïve (*n* = 2) and previously treated (*n* = 11) Japanese patients, respectively (28). The efficacy of tepotinib in Japanese patients presented here is consistent with previously reported outcomes for the overall VISION study population (15), in which tepotinib demonstrated consistent efficacy irrespective of the number of prior lines of therapy received.

In line with the overall population in the VISION study, tepotinib was well tolerated in Japanese patients, with mostly mild-to-moderate AEs and a low rate of treatment-related AEs leading to treatment discontinuation. Peripheral oedema, which is considered a class effect of MET inhibitors, was common but considered manageable and did not lead to permanent treatment discontinuation in any Japanese patients. Of note, blood creatinine increase was the most common treatment-related AE experienced in Japanese patients (63.2%, all Grade 1–2). Blood creatinine increase was also the most common treatment-related AE in Japanese patients in the GEOMETRY mono-1 study of capmatinib (28). Based on non-clinical studies, increases in creatinine may reflect direct inhibitory effects on renal tubular transporters (29,30). Although low-grade interstitial lung disease was reported in two Japanese patients, the events were managed with treatment interruptions and dose reductions, enabling these patients to continue to benefit from treatment for >7 months. Exacerbations of interstitial lung disease during treatment for lung cancer have been observed with increased frequency in Japanese patients (31) and, because it can be fatal, there is a need for lung function monitoring in patients being treated for NSCLC.

Although the number of Japanese patients analysed for efficacy is limited (*n* = 15), results suggest a robust clinical benefit of tepotinib in Japanese patients during a median of 18 months of follow-up for survival.

## Conclusions

In conclusion, our findings indicate that the selective MET inhibitor tepotinib demonstrated durable clinical benefit in Japanese patients with advanced NSCLC harbouring *MET* exon 14 skipping. Tepotinib was well tolerated, with mostly mild-to-moderate AEs, and few treatment discontinuations. Results from the VISION study led to regulatory approval of tepotinib and its companion diagnostic assay for the detection of *MET* alterations (ArcherMET CDx) in March 2020 in Japan. The high response rate of tepotinib in patients with *MET* exon 14 skipping highlights the importance of molecular testing during NSCLC diagnosis and indicates a potentially significant role for liquid biopsies in terms of indicating patients who could benefit from tepotinib.

## Supplementary Material

JJCO_2021_VISION_Japanese_subset_Supplemental_Figure_S1_hyab072
