# Supplementary figures and images for: Tepotinib in patients with NSCLC harbouring MET exon 14 skipping: Japanese subset analysis from the Phase II VISION study
Source: Jpn J Clin Oncol. 2021 May 25;51(8):1261–8. doi: 10.1093/jjco/hyab072 (PMC8326385; doi:10.1093/jjco/hyab072)

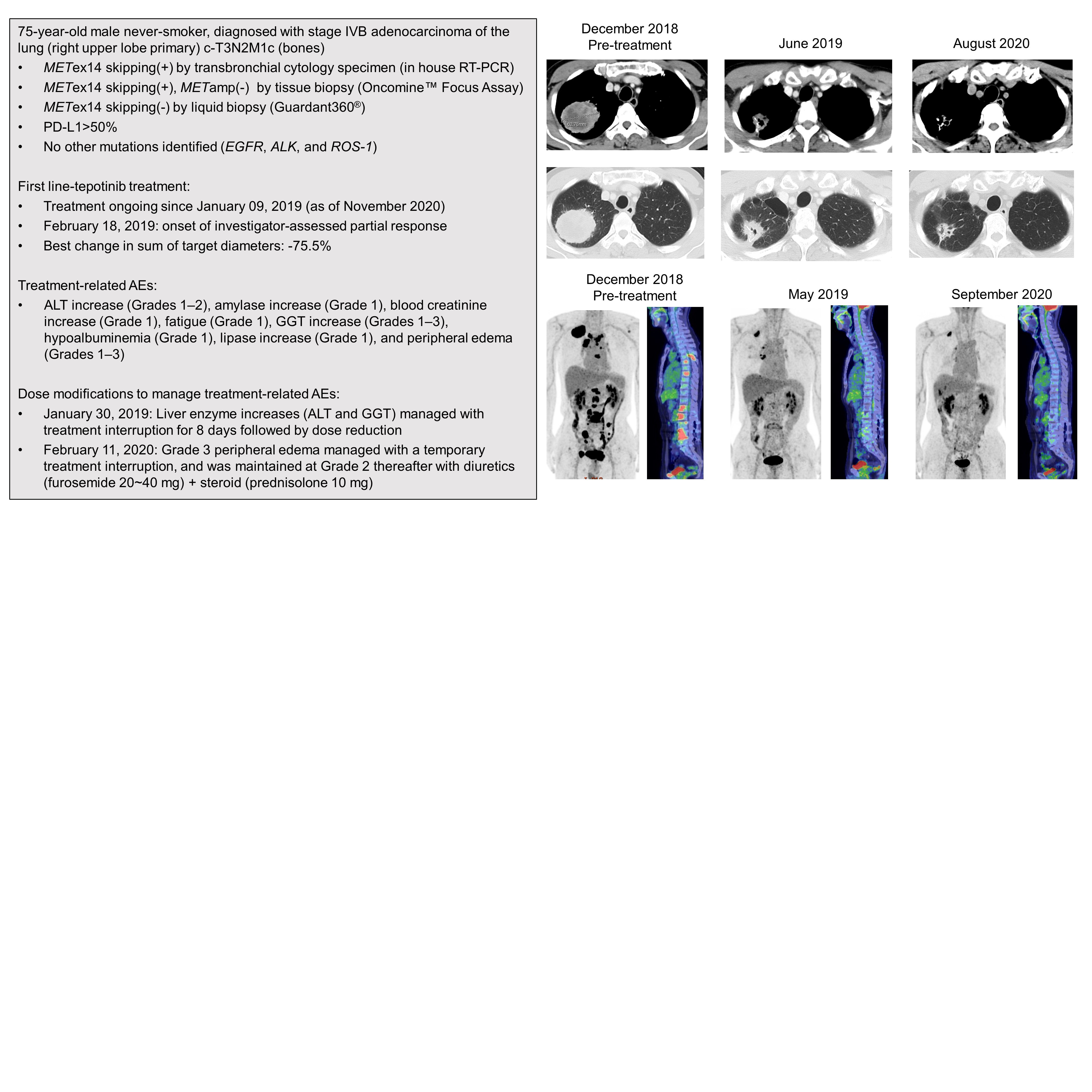

Supplement: JJCO_2021_VISION_Japanese_subset_Supplemental_Figure_S1_hyab072 [file jjco_2021_vision_japanese_subset_supplemental_figure_s1_hyab072.jpeg]
